# 2,4-Dimethoxy­benzaldehyde azine

**DOI:** 10.1107/S1600536809038082

**Published:** 2009-09-26

**Authors:** M. A. A. A. A. Islam, M. T. H. Tarafder, M. A. Alam, N. Guidolin, E. Zangrando

**Affiliations:** a Department of Chemistry, Rajshahi University of Engineering and Technology, Rajshahi 6204, Bangladesh; b Department of Chemistry, Rajshahi University, Rajshahi 6205, Bangladesh; c Dipartimento di Scienze Chimiche, Via Licio Giorgieri 1, 34127 Trieste, Italy

## Abstract

The title mol­ecule, C_18_H_20_N_2_O_4_, is located on a crystallographic centre of symmetry. The meth­oxy groups are coplanar with the benzene ring [interplanar angles of 14.4 (2) and 3.1 (3)°], indicating a conjugation effect.

## Related literature

For the structures of related compounds, see: Narayana *et al.* (2007 ▶); Liu *et al.* (2007 ▶); Takakashi *et al.* (2006 ▶). For a statistical study of meth­oxy groups bound to phenyl rings, see: Hummel *et al.* (1988 ▶).
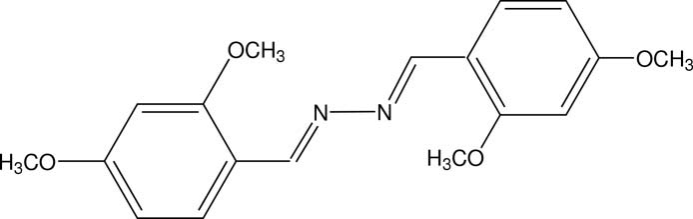


## Experimental

### 

#### Crystal data

C_18_H_20_N_2_O_4_
*M* *_r_* = 328.36
Monoclinic, 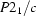

*a* = 6.775 (2) Å
*b* = 9.014 (3) Å
*c* = 14.081 (3) Å
β = 100.42 (2)°
*V* = 845.7 (4) Å^3^
*Z* = 2
Mo *K*α radiation
μ = 0.09 mm^−1^
*T* = 293 K
0.45 × 0.30 × 0.12 mm

#### Data collection

Enraf–Nonius DIP1030 image-plate diffractometer
Absorption correction: none
3535 measured reflections
1293 independent reflections
719 reflections with *I* > 2σ(*I*)
*R*_int_ = 0.058
θ_max_ = 24.1°

#### Refinement

*R*[*F* ^2^ > 2σ(*F* ^2^)] = 0.045
*wR*(*F* ^2^) = 0.121
*S* = 0.84
1293 reflections
110 parameters
H-atom parameters constrained
Δρ_max_ = 0.12 e Å^−3^
Δρ_min_ = −0.10 e Å^−3^

### 

Data collection: *XPRESS* (MacScience, 2002 ▶; cell refinement: *DENZO* (Otwinowski & Minor, 1997 ▶); data reduction: *DENZO* and *SCALEPACK* (Otwinowski & Minor, 1997 ▶); program(s) used to solve structure: *SHELXS97* (Sheldrick, 2008 ▶); program(s) used to refine structure: *SHELXL97* (Sheldrick, 2008 ▶); molecular graphics: *ORTEP-3 for Windows* (Farrugia, 1997 ▶); software used to prepare material for publication: *WinGX* (Farrugia, 1999 ▶).

## Supplementary Material

Crystal structure: contains datablocks global, I. DOI: 10.1107/S1600536809038082/rz2359sup1.cif

Structure factors: contains datablocks I. DOI: 10.1107/S1600536809038082/rz2359Isup2.hkl

Additional supplementary materials:  crystallographic information; 3D view; checkCIF report

## Figures and Tables

**Fig. 1.**
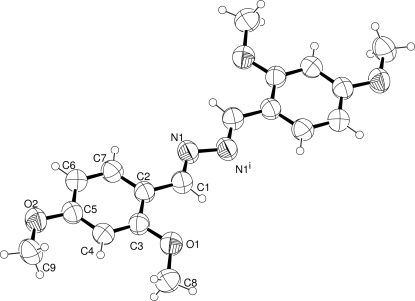
ORTEP drawing (ellipsoids at the 40% probability level) of the title compound with atom labelling scheme. Primed atom at -x - 1, -y + 1, -z + 1.

## supplementary crystallographic information

### Comment

The molecule of the title compound (Fig. 1) possesses a crystallographically imposed inversion centre at the midpoint of the N—N bond. Thus the conformation about the N1—N1^i^ azine bridge (symmetry code: (i) -*x* - 1, -*y* + 1, -*z* + 1) is *trans*, with a torsion angle C1—N1—N1'—C1' of 180°. The molecule has coplanar non hydrogen atoms as found in other similar structures (Narayana *et al.*, 2007; Takakashi *et al.*, 2006). The coplanarity of methoxy groups with the benzene ring suggests the presence of a conjugation effect (Hummel *et al.*, 1988). The bond distance N1—N1^i^ of 1.414 (4) Å, is comparable to those measured in the structure of methoxybenzaldehyde azine and *N*,*N*'-bis(4-hydroxybenzylidene)-hydrazine, which are 1.418 and 1.415 Å, respectively (Narayana *et al.*, 2007; Liu *et al.*, 2007). The crystal packing does not show any π-π stacking interactions between aromatic rings.

### Experimental

Hydrazine hydrate (99.99%) (0.13 g, 2.5 mmol) was added to a solution of 2,4-dimethoxybenzaldehyde (0.83 g, 5 mmol) in methanol (10 ml) with constant stirring for 5 min, at room temperature. A light yellow precipitate was formed, which was separated by filtration, washed with methanol and dried at 70°C for 1 h and then preserved *in vacuo* over anhydrous CaCl_2_ (0.43 g, 44.79%, m.p. 473 K). The compound was soluble in chloroform, benzene and toluene whereas insoluble in acetonitrile, methanol and ethanol. The compound (0.37 g) was dissolved in chloroform (60 ml) at boiling temperature and allowed to stand at room temperature (20–25°C). Bright large yellow crystals, suitable for X-ray single-crystal analysis, were obtained after 7 days. The crystals were collected, washed with absolute ethanol and dried *in vacuo* over anhydrous CaCl_2_.

### Refinement

All H atoms were located geometrically and treated as riding atoms, with C—H = 0.93–0.96 Å, and with *U*_iso_(H) = 1.2*U*_eq_(C) or 1.5*U*_eq_(C) for methyl H atoms.

### Crystal data

**Table:**

C_18_H_20_N_2_O_4_	*F*(000) = 348
*M**_r_* = 328.36	*D*_x_ = 1.289 Mg m^−^^3^
Monoclinic, *P*2_1_/*c*	Melting point: 475 K
Hall symbol: -P 2ybc	Mo *K*α radiation, λ = 0.71073 Å
*a* = 6.775 (2) Å	Cell parameters from 482 reflections
*b* = 9.014 (3) Å	θ = 3.6–20.7°
*c* = 14.081 (3) Å	µ = 0.09 mm^−^^1^
β = 100.42 (2)°	*T* = 293 K
*V* = 845.7 (4) Å^3^	Plate, yellow
*Z* = 2	0.45 × 0.30 × 0.12 mm

### Data collection

**Table:**

Enraf–Nonius DIP1030 image-plate diffractometer	719 reflections with *I* > 2σ(*I*)
Radiation source: fine-focus sealed tube	*R*_int_ = 0.058
graphite	θ_max_ = 24.1°, θ_min_ = 2.9°
φ–scans with narrow frames	*h* = −7→7
3535 measured reflections	*k* = −10→10
1293 independent reflections	*l* = −16→16

### Refinement

**Table:**

Refinement on *F*^2^	Secondary atom site location: difference Fourier map
Least-squares matrix: full	Hydrogen site location: inferred from neighbouring sites
*R*[*F*^2^ > 2σ(*F*^2^)] = 0.045	H-atom parameters constrained
*wR*(*F*^2^) = 0.121	*w* = 1/[σ^2^(*F*_o_^2^) + (0.0678*P*)^2^] where *P* = (*F*_o_^2^ + 2*F*_c_^2^)/3
*S* = 0.84	(Δ/σ)_max_ < 0.001
1293 reflections	Δρ_max_ = 0.12 e Å^−^^3^
110 parameters	Δρ_min_ = −0.10 e Å^−^^3^
0 restraints	Extinction correction: *SHELXL97* (Sheldrick, 2008), Fc^*^=kFc[1+0.001xFc^2^λ^3^/sin(2θ)]^-1/4^
Primary atom site location: structure-invariant direct methods	Extinction coefficient: 0.070 (13)

### Special details

**Table:**

Geometry. All s.u.'s (except the s.u. in the dihedral angle between two l.s. planes) are estimated using the full covariance matrix. The cell s.u.'s are taken into account individually in the estimation of s.u.'s in distances, angles and torsion angles; correlations between s.u.'s in cell parameters are only used when they are defined by crystal symmetry. An approximate (isotropic) treatment of cell s.u.'s is used for estimating s.u.'s involving l.s. planes.
Refinement. Refinement of *F*^2^ against ALL reflections. The weighted *R*-factor *wR* and goodness of fit *S* are based on *F*^2^, conventional *R*-factors *R* are based on *F*, with *F* set to zero for negative *F*^2^. The threshold expression of *F*^2^ > 2σ(*F*^2^) is used only for calculating *R*-factors(gt) *etc*. and is not relevant to the choice of reflections for refinement. *R*-factors based on *F*^2^ are statistically about twice as large as those based on *F*, and *R*- factors based on ALL data will be even larger.

### Fractional atomic coordinates and isotropic or equivalent isotropic displacement parameters (Å^2^)

**Table:**

	*x*	*y*	*z*	*U*_iso_*/*U*_eq_	
N1	−0.4202 (3)	0.4904 (2)	0.53948 (13)	0.0857 (6)	
O1	0.0297 (2)	0.77203 (18)	0.52320 (12)	0.0960 (6)	
O2	0.4398 (2)	0.55904 (18)	0.80720 (11)	0.0952 (6)	
C1	−0.2709 (4)	0.5738 (3)	0.53259 (16)	0.0833 (7)	
H1	−0.2800	0.6376	0.4800	0.100*	
C2	−0.0880 (3)	0.5722 (2)	0.60384 (15)	0.0745 (6)	
C3	0.0667 (3)	0.6732 (2)	0.59790 (16)	0.0768 (6)	
C4	0.2433 (3)	0.6732 (2)	0.66487 (16)	0.0785 (7)	
H4	0.3431	0.7425	0.6606	0.094*	
C5	0.2699 (3)	0.5695 (3)	0.73791 (16)	0.0787 (6)	
C6	0.1196 (4)	0.4673 (3)	0.74624 (17)	0.0850 (7)	
H6	0.1372	0.3984	0.7962	0.102*	
C7	−0.0547 (3)	0.4704 (3)	0.67925 (18)	0.0830 (7)	
H7	−0.1546	0.4017	0.6845	0.100*	
C8	0.1918 (4)	0.8551 (3)	0.49880 (19)	0.1041 (9)	
H8A	0.1429	0.9185	0.4449	0.156*	
H8B	0.2905	0.7885	0.4820	0.156*	
H8C	0.2514	0.9144	0.5531	0.156*	
C9	0.5936 (3)	0.6675 (3)	0.80431 (19)	0.1042 (9)	
H9A	0.7046	0.6488	0.8557	0.156*	
H9B	0.5410	0.7648	0.8118	0.156*	
H9C	0.6381	0.6614	0.7435	0.156*	

### Atomic displacement parameters (Å^2^)

**Table:**

	*U*^11^	*U*^22^	*U*^33^	*U*^12^	*U*^13^	*U*^23^
N1	0.0716 (13)	0.0972 (13)	0.0854 (14)	0.0055 (12)	0.0066 (9)	−0.0007 (11)
O1	0.0858 (11)	0.1004 (12)	0.0995 (12)	−0.0022 (9)	0.0106 (9)	0.0169 (10)
O2	0.0820 (11)	0.1060 (13)	0.0915 (12)	−0.0027 (9)	−0.0012 (9)	0.0038 (9)
C1	0.0778 (16)	0.0904 (16)	0.0803 (15)	0.0037 (13)	0.0101 (13)	0.0021 (12)
C2	0.0693 (14)	0.0799 (14)	0.0732 (14)	0.0017 (12)	0.0099 (12)	−0.0017 (12)
C3	0.0782 (15)	0.0787 (15)	0.0728 (14)	0.0082 (13)	0.0118 (13)	0.0026 (12)
C4	0.0734 (15)	0.0799 (15)	0.0818 (15)	−0.0017 (12)	0.0132 (13)	−0.0029 (13)
C5	0.0722 (15)	0.0864 (15)	0.0755 (15)	0.0053 (13)	0.0084 (13)	−0.0065 (13)
C6	0.0806 (16)	0.0919 (16)	0.0815 (16)	0.0009 (13)	0.0114 (14)	0.0056 (13)
C7	0.0772 (16)	0.0870 (16)	0.0841 (16)	−0.0032 (12)	0.0126 (13)	−0.0010 (13)
C8	0.1061 (19)	0.0956 (17)	0.113 (2)	−0.0067 (16)	0.0255 (16)	0.0164 (15)
C9	0.0775 (16)	0.115 (2)	0.114 (2)	−0.0073 (15)	0.0006 (15)	−0.0033 (16)

### Geometric parameters (Å, °)

**Table:**

N1—C1	1.278 (3)	C4—H4	0.9300
N1—N1^i^	1.414 (4)	C5—C6	1.393 (3)
O1—C3	1.366 (2)	C6—C7	1.372 (3)
O1—C8	1.422 (3)	C6—H6	0.9300
O2—C5	1.371 (3)	C7—H7	0.9300
O2—C9	1.435 (3)	C8—H8A	0.9600
C1—C2	1.446 (3)	C8—H8B	0.9600
C1—H1	0.9300	C8—H8C	0.9600
C2—C7	1.391 (3)	C9—H9A	0.9600
C2—C3	1.402 (3)	C9—H9B	0.9600
C3—C4	1.383 (3)	C9—H9C	0.9600
C4—C5	1.378 (3)		
			
C1—N1—N1^i^	111.8 (2)	C7—C6—C5	118.7 (2)
C3—O1—C8	119.13 (18)	C7—C6—H6	120.7
C5—O2—C9	116.98 (19)	C5—C6—H6	120.7
N1—C1—C2	122.2 (2)	C6—C7—C2	122.6 (2)
N1—C1—H1	118.9	C6—C7—H7	118.7
C2—C1—H1	118.9	C2—C7—H7	118.7
C7—C2—C3	117.0 (2)	O1—C8—H8A	109.5
C7—C2—C1	122.4 (2)	O1—C8—H8B	109.5
C3—C2—C1	120.5 (2)	H8A—C8—H8B	109.5
O1—C3—C4	122.7 (2)	O1—C8—H8C	109.5
O1—C3—C2	115.7 (2)	H8A—C8—H8C	109.5
C4—C3—C2	121.5 (2)	H8B—C8—H8C	109.5
C5—C4—C3	119.3 (2)	O2—C9—H9A	109.5
C5—C4—H4	120.4	O2—C9—H9B	109.5
C3—C4—H4	120.4	H9A—C9—H9B	109.5
O2—C5—C4	123.9 (2)	O2—C9—H9C	109.5
O2—C5—C6	115.3 (2)	H9A—C9—H9C	109.5
C4—C5—C6	120.8 (2)	H9B—C9—H9C	109.5
			
N1^i^—N1—C1—C2	−178.8 (2)	C2—C3—C4—C5	1.5 (3)
N1—C1—C2—C7	6.4 (3)	C9—O2—C5—C4	2.6 (3)
N1—C1—C2—C3	−175.1 (2)	C9—O2—C5—C6	−176.66 (18)
C8—O1—C3—C4	15.1 (3)	C3—C4—C5—O2	179.32 (18)
C8—O1—C3—C2	−166.0 (2)	C3—C4—C5—C6	−1.5 (3)
C7—C2—C3—O1	−179.90 (18)	O2—C5—C6—C7	−179.73 (19)
C1—C2—C3—O1	1.5 (3)	C4—C5—C6—C7	1.0 (3)
C7—C2—C3—C4	−1.0 (3)	C5—C6—C7—C2	−0.5 (3)
C1—C2—C3—C4	−179.62 (19)	C3—C2—C7—C6	0.5 (3)
O1—C3—C4—C5	−179.68 (18)	C1—C2—C7—C6	179.1 (2)

Symmetry codes: (i) −*x*−1, −*y*+1, −*z*+1.
